# Simultaneous electrochemical determination of uric acid and hypoxanthine at a TiO_2_/graphene quantum dot-modified electrode

**DOI:** 10.3762/bjnano.15.60

**Published:** 2024-06-20

**Authors:** Vu Ngoc Hoang, Dang Thi Ngoc Hoa, Nguyen Quang Man, Le Vu Truong Son, Le Van Thanh Son, Vo Thang Nguyen, Le Thi Hong Phong, Ly Hoang Diem, Kieu Chan Ly, Ho Sy Thang, Dinh Quang Khieu

**Affiliations:** 1 University of Sciences, Hue University, Vietnam; 2 Tan Hiep High School, Kien giang, Vietnam; 3 University of Medicine and Pharmacy, Hue University, Vietnam; 4 The University of Danang, University of Science and Education, Vietnam; 5 Institute of Materials Science, Vietnam Academy of Science and Technology, Vietnam; 6 Kien Giang University, Vietnam; 7 Dong Thap University, Vietnam

**Keywords:** graphene quantum dots, hypoxanthine, peroxo titanium complexes, uric acid

## Abstract

A TiO_2_/graphene quantum dots composite (TiO_2_/GQDs) obtained by in situ synthesis of GQDs, derived from coffee grounds, and peroxo titanium complexes was used as electrode modifier in the simultaneous electrochemical determination of uric acid and hypoxanthine. The TiO_2_/GQDs material was characterized by photoluminescence, X-ray diffraction, Raman spectroscopy, high-resolution transmission electron microscopy, and energy-dispersive X-ray mapping. The TiO_2_/GQDs-GCE exhibits better electrochemical activity for uric acid and hypoxanthine than GQDs/GCE or TiO_2_/GCE in differential pulse voltammetry (DPV) measurements. Under optimized conditions, the calibration plots were linear in the range from 1.00 to 15.26 μM for both uric acid and hypoxanthine. The limits of detection of this method were 0.58 and 0.68 μM for uric acid and hypoxanthine, respectively. The proposed DPV method was employed to determine uric acid and hypoxanthine in urine samples with acceptable recovery rates.

## Introduction

Uric acid (7,9-dihydro-1*H*-purine-2,6,8(3*H*)-trione, URI) is the primary final product of the purine metabolism in humans. High concentrations of URI in serum and urine are a sign of several diseases, such as gout, Lesch–Nyan disease, obesity, diabetes, high cholesterol levels, and kidney diseases [1–2]. Hypoxanthine (6-hydroxypurine, HYP), a purine derivative, is an indicator of hypoxia [3]. A lower HYP concentration in the cerebrospinal fluid of patients compared to healthy individuals is related to Parkinson’s disease [4]. The oxidation of HYP yields xanthine, which can be further oxidized to URI. Meat from shrimp, fish, and some other animals contain large amounts of HYP. High consumption of these foods has been considered to result in elevated levels of URI, associated with the risk of gout [5]. Hence, simple and selective methods to determine the purine content in food and dietary products are of interest among researchers [6–7]. At the present time, several techniques for the determination of URI and HYP are available, such as high-performance liquid chromatography [8], liquid chromatography–tandem mass spectrometry [9], and gas chromatography/mass spectrometry [10]. These approaches require complex handling procedures. Electrochemical techniques have been considered as a robust alternative for URI and HYP sensing because of their simplicity, cost-effectiveness, and reliability. The use of modified electrodes has shown to improve significantly electrocatalytic activity and electrical conductivity of electrodes.

Graphene, with sp^2^-hybridized carbon atoms in a single layer, has gained much attention because of its unique physicochemical properties. Graphene quantum dots (GQDs) are zero-dimensional graphene derivatives consisting of one to few layers of graphene sheets with a size of less than 20 nm in width [11]. The missing bandgap results in an absence of luminescence in pristine graphene. However, a bandgap can be generated in GQDs through edge effects. Edge-functionalized GQDs have oxygen-containing functional groups such as hydroxy, carboxyl, carbonyl, and epoxy groups, which can conjugate to various biological/organic/inorganic molecules such as proteins, antibodies, or metal ions [12]. The capability of electron transfer/energy storage derived from their conjugate structure makes them effectively utilizable over the full light spectrum [13–14]. GQDs can be prepared through solvothermal/hydrothermal processes or carbonization from suitable organic molecules (polymers or biomass) [15]. Biomass waste (e.g., agricultural residues and food waste) is a renewable resource for the preparation of high-value carbon materials. Among food wastes, coffee grounds are a promising precursor to prepare GQDs with new functionalities regarding a more sustainable materials industry [16]. Considering the fields of catalysis and electrochemistry, combining GQDs with semiconductors, especially TiO_2_, has been of interest. For example, GQDs have been successfully introduced into TiO_2_ [17] to enhance its photocatalytic activity. John Peter et al. reported TiO_2_/GQDs as anodes for enhancing the short-circuit current in solar cells [18]. The lithium-ion storage performance of C-GQDs/α-Fe_2_O_3_ composites in lithium batteries presented excellent cyclability and rate capability [19]. Among several chemical approaches for the synthesis of titania, sol–gel processes have been commonly used for practical application. In these sol–gel methods, titanium alkoxides and halides are extensively used as precursors [20–21]. Because of their high reactivity, a complicate control over the reaction conditions is critical to achieve the desired crystalline structures and morphology [22]. Recently, a synthesis using stable water-soluble titanium complexes has been developed to overcome the disadvantages of these precursors and to easily obtain fine titania particles with a controlled shape [23]. The synthesis of TiO_2_/GQDs from water-soluble titanium complexes is expected to form a homogeneous suspension that is convenient for developing modified electrodes used in electrochemical analysis. To the best of our knowledge, there are only few reports on TiO_2_/GQDs for electrochemical analysis.

In the present work, TiO_2_/GQDs were prepared from coffee grounds and peroxo titanium complexes by a hydrothermal process. The simultaneous determination of URI and HYP using a TiO_2_/GQDs-modified electrode were addressed.

## Experimental

### Materials

Coffee grounds were collected from the local area. Anatase (98%), hydrogen chloride (39%), hydrogen peroxide (30%), boric acid (99%), phosphoric acid (85%), acetic acid (99%), uric acid (99%), and hypoxanthine (99%) were provided by the Merck company, Germany. Stock solutions of URI and HYP were prepared prior to use.

### Equipment

The X-ray diffraction patterns of the suspensions of GQDs, peroxo titanium complexes, and TiO_2_/GQDs were recorded on a D8 Advance, Bruker, Germany with background subtraction. The morphologies of GQDs were observed by using a JEM 2100 high-resolution transmission electron microscopy (HRTEM), Joel, Japan. Raman spectroscopy measurements were performed on a WiTec, Alpha 300R with a 532 nm laser. Surface analyses of the obtained materials were carried out using a S-4800 scanning electron microscope (SEM), Hitachi (Japan). UV–vis absorption spectroscopy measurements was carried out on an 8453 UV–vis spectrophotometer, Agilent, USA. Photoluminescence (PL) spectroscopy measurements were performed on a FL3C-22 spectrophotometer, Horiba, USA.

### Synthesis of TiO_2_/GQDs

Peroxo titanium complexes were synthesized in a hydrothermal process. 0.25 g of TiO_2_ and 12.5 mL of 10 M NaOH were mixed under ultrasonic stirring. The suspension was then transferred to a teflon-lined stainless steel autoclave and heated to 130 °C for 10 h. The resulting white solid was separated by centrifugation and rinsed several times with 0.1 M HCl and distilled water until a supernatant with neutral pH was obtained. The solid was dried at 80 °C for 2 h. This product was then mixed with 30 mL of H_2_O_2_ (35%, *d* = 1.11 g·cm^3^) at 90 °C under magnetic stirring for 1 h to obtain a clear yellow solution of peroxo titanium complexes. The concentration of TiO_2_ in the complexes was found as 0.745% (w/w) (0.25 g of TiO_2_ in 30 mL of H_2_O_2_).

GQDs were synthesized from coffee grounds. Coffee grounds (0.2 g) were first suspended in water (50 mL). The suspension was sonicated for 1 h before being transferred to the autoclave and heated up to 180 °C for 10 h. The resulting product was then cooled to room temperature and separated by centrifugation at 4000 rpm to obtain a dark brown solid. The concentration of GQDs calculated after subtracting the insoluble part was 0.249% (w/w) (0.125 g of coffee grounds in 50 mL of water).

TiO_2_/GQDs suspensions with different TiO_2_/GQDs ratios were prepared by mixing different volumes of GQDs and peroxo titanium complex suspensions under stirring at 50 °C for 1 h. The samples are denoted as (0:4)TiO_2_/GQDs; (1:4)TiO_2_/GQDs; (2:4)TiO_2_/GQDs; (4:4)TiO_2_/GQDs; (4:2)TiO_2_/GQDs; (4:1)TiO_2_/GQDs; (4:0)TiO_2_/GQDs. The numbers in parentheses stand for the volume ratio of peroxo titanium complex to GQDs solutions.

### Electrochemical studies

Voltammetric measurements were performed at room temperature using a CPA-HH5 electrochemical workstation, Vietnam. A conventional three-electrode cell with a glassy carbon working electrode (GCE, 3 mm diameter), a platinum wire auxiliary electrode, and a Ag/AgCl reference electrode was used. Electrolyte solutions were prepared using doubly distilled water. Britton**–**Robinson (BR) buffer solutions with pH between 2 and 6 were prepared by mixing boric acid solution, phosphoric acid solution, and acetic acid solution in appropriate ratios.

To prepare a TiO_2_/GQDs-modified glassy carbon electrode (TiO_2_/GQDs-GCE), 5 mL of TiO_2_/GQDs suspension in water (0.1 mg/mL) was cast onto the surface of the bare GCE. The modified electrode was allowed to dry naturally for some hours at ambient temperature.

The urine samples for electrochemical measurement were prepared by first diluting 1 mL urine to 20 mL with distilled water. 1.5 mL of this urine solution was added to 2 mL of 0.25 M pH 3 buffer and was diluted to 10 mL with distilled water in an electrolysis cell. The solution was spiked with 10 µL of 500 µM URI and 20 µL of 500 µM HYT before dilution to 10 mL.

## Results and Discussion

### Characterization of materials

**Figure 1:**
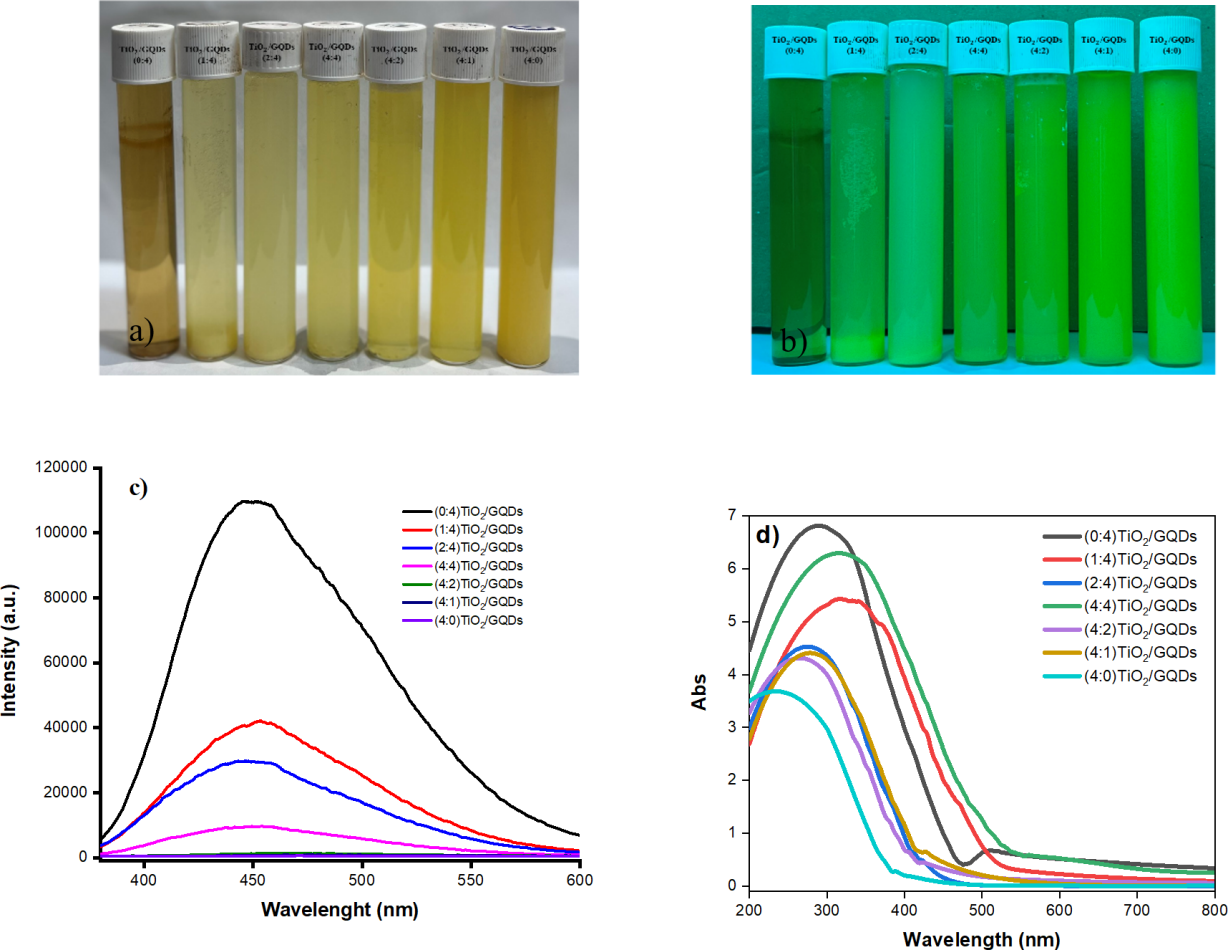
Images of aqueous suspension of TiO_2_/GQDs samples (a) under white light and (b) under UV light. (c) Photoluminescence spectra excited at a wavelength of 365 nm. (d) UV–vis adsorption spectra of TiO_2_, GQDs, and TiO_2_/GQDs suspensions.

The mixtures of GQDs and TiO_2_/GQDs suspensions were exposed to visible and UV light to confirm their fluorescence behavior. Under visible light, the aqueous suspension of GQDs is transparent and brown in color, while the suspension of the peroxo titanium complex is opaque yellow. The prepared TiO_2_/GQD aqueous suspensions, in contrast, display a light yellow color, which gets darker as the ratio of TiO_2_/GQD in the complex increases (Figure 1a). Under 365 nm UV irradiation (Figure 1b), the GQDs displays green luminescence, while the TiO_2_/GQDs samples exhibit different levels of fluorescence. Figure 1c presents the emission spectra of GQDs and TiO_2_/GQDs. GQDs exhibits an emission maximum at 450 nm corresponding to the blue luminescence. The luminous intensity at an emission wavelength of ca. 450 nm decreases with increasing ratio of TiO_2_/GQDs in the material until it almost disappears when the fraction of GQDs is lower than that of TiO_2_. This indicates that mainly GQDs contribute to the luminescence of all samples. Figure 1d presents the UV–vis spectra of the obtained suspensions. The UV–vis spectra show absorbance bands from 200–400 nm that are typical for the colloidal systems with nanoscale particles [24–25]. The UV–vis spectrum of GQDs shows a characteristic feature of GO at ≈300 nm due to the absorption of the graphitic structure [26], while that of titanium complexes presents an absorption band at 240 nm. The combination of titanium complexes with GQDs resulted in higher adsorption λ_max_ values.

**Figure 2:**
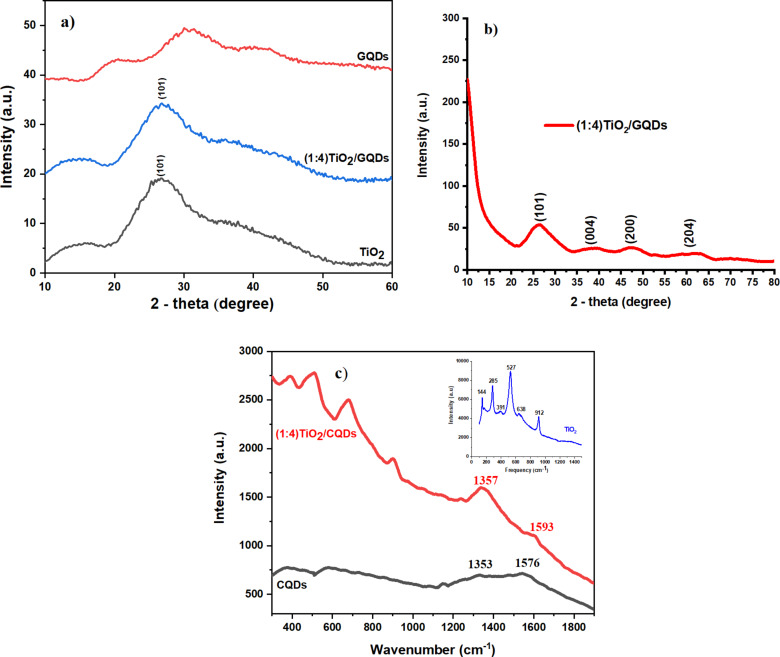
(a) XRD patterns of TiO_2_, GQDs, and (1:4)TiO_2_/GQDs in suspension form. (b) XRD pattern of (1:4)TiO_2_/GQDs in solid form. (c) Raman spectra of TiO_2_, GQDs, and (1:4)TiO_2_/GQDs in solid form.

XRD patterns of the obtained samples in suspension form are presented in Figure 2. The XRD patterns of TiO_2_ and TiO_2_/GQDs suspensions show broad peaks of TiO_2_ at 2θ = 26°, corresponding to the (101) plane of the anatase phase (JCPDS file 73-1764) (Figure 2a). As the XRD measurements were made in liquids instead of solid powders, the diffraction peaks are found to be broad and weak. The diffraction peaks of the GQDs at 2θ = 30.4° can be assigned to the (002) plane of graphene. The broad nature of the diffraction peak is due to the structure of GQDs containing only few layers of graphene sheets [27]. The XRD pattern of TiO_2_/GQDs in solid form (dried at 100 °C for 3 h) exhibits characteristic peaks of anatase at 2θ = 25.6°, 38.1°, and 48.3°, corresponding to the (101), (004), and (200) planes, respectively. This confirms that the (1:4)TiO_2_/GQDs suspension contains fine anatase nanoparticles. The broadening of XRD peaks in suspension was employed to evaluate the size (*D*_hkl_) of the crystal domains. It was calculated from the full width at half maximum (*B*) using the Scherrer equation 
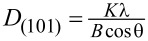
, where λ is the X-ray wavelength (1.5406 Å), θ is the Bragg angle, and *K* is a constant (ca. 0.9). The average crystallite size of the synthesized TiO_2_ nanoparticles was found to be 7.1 nm. It is notable that no typical peaks for GQDs can be found in the XRD pattern of solid TiO_2_/GQDs. This is possibly because of the low content of GQDs in the composites, which clearly indicates that GQDs do not affect significantly TiO_2_ crystal structure.

Figure 2c presents the Raman spectra of the obtained materials. Four characteristic Raman-active E_g_, B1_g_, A1_g_, and E_g_ modes of anatase TiO_2_ are seen at 144, 395, 516, and 639 cm^−1^, respectively [28]. In the Raman spectrum of GQDs, the peak at 1353 cm^−1^ can be attributed to the D band, which can be assigned to the vibrations of carbon atoms because of the presence of structural defects. The peak at 1576 cm^−1^ can be assigned to the G band due to the vibrations of sp^2^-hybridized carbon atoms in graphene. The ratio of *I*_D_/*I*_G_ is characteristic for the disorder of the graphene structure [29]. The characteristic vibrations for anatase are also observed in the Raman spectrum of the TiO_2_/GQDs sample. However, the D and G bands of GQDs shift to 1357 and 1593 cm^−1^, respectively, in the TiO_2_/GQDs spectrum. The difference in *I*_D_/*I*_G_ ratio, 0.94 for GQDs and 0.71 for TiO_2_/GQDs, may be due to the interaction between TiO_2_ and GQDs.

**Figure 3:**
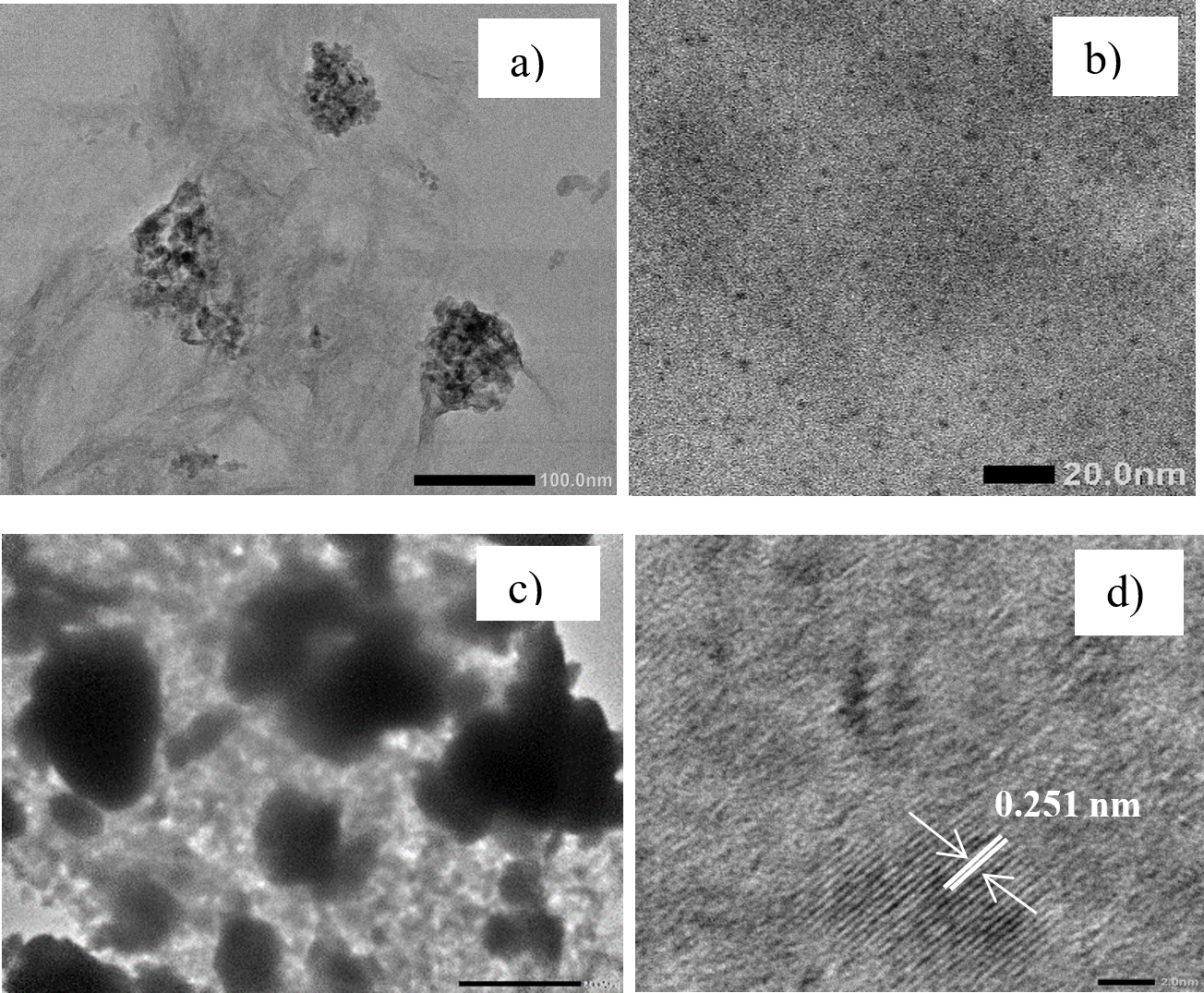
HRTEM images of (a) TiO_2_, (b) GQDs, (c) (1:4)TiO_2_/GQDs, and (d) of (1:4)TiO_2_/GQDs with high magnification.

Figure 3 presents TEM observations of the obtained materials. The morphology of TiO_2_ shows agglomerates of around 50–80 nm that consist of fine nanoparticles (Figure 3a), while that of GQDs shows fine spherical particles around 3–5 nm with high depression. In the composite sample, TiO_2_ nanoparticles of around 100 nm can be seen to be highly dispersed in the GQDs matrix (Figure 3c). The TiO_2_ particles in TiO_2_/GQDs in Figure 3d were identified by HRTEM with an interplanar distance of (101) planes of 0.251 nm, which has also been revealed by XRD of TiO_2_ in anatase form.

**Figure 4:**
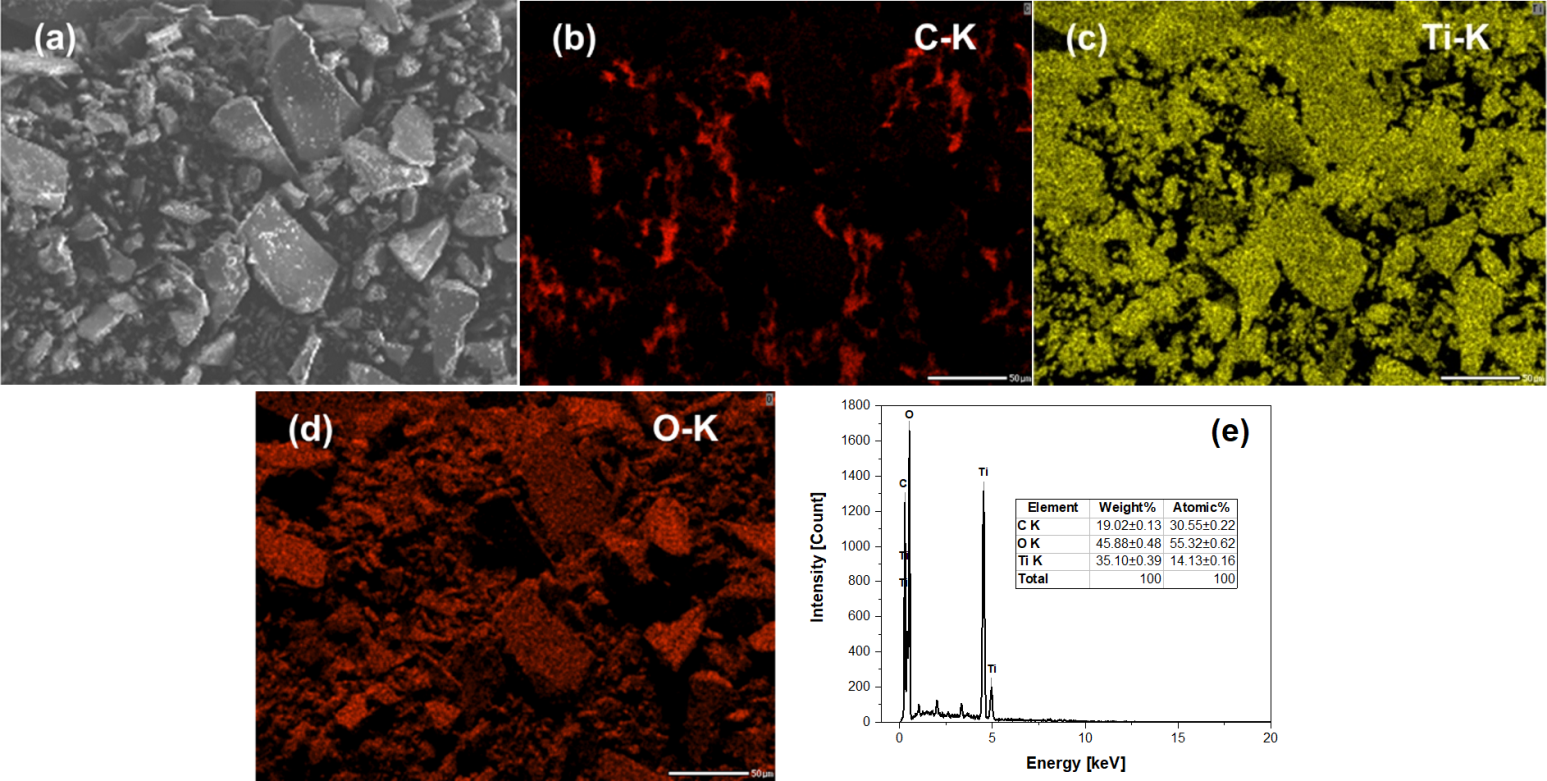
EDX mapping of (1:4)TiO_2_/GQDs.

To further determine the composition of the prepared TiO_2_/GQDs composites, EDX mapping was used (Figure 4). The obtained results reveal that TiO_2_/GQDs contain C (30.6%), O (55.3%), and Ti (14.1%), evenly distributed across the sample. This is in agreement with the XRD, Raman, and HRTEM observations.

### Simultaneous voltammetric determination of uric acid (URI) and hypoxanthine (HYP)

#### Cyclic voltammetric behavior of different electrodes

**Figure 5:**
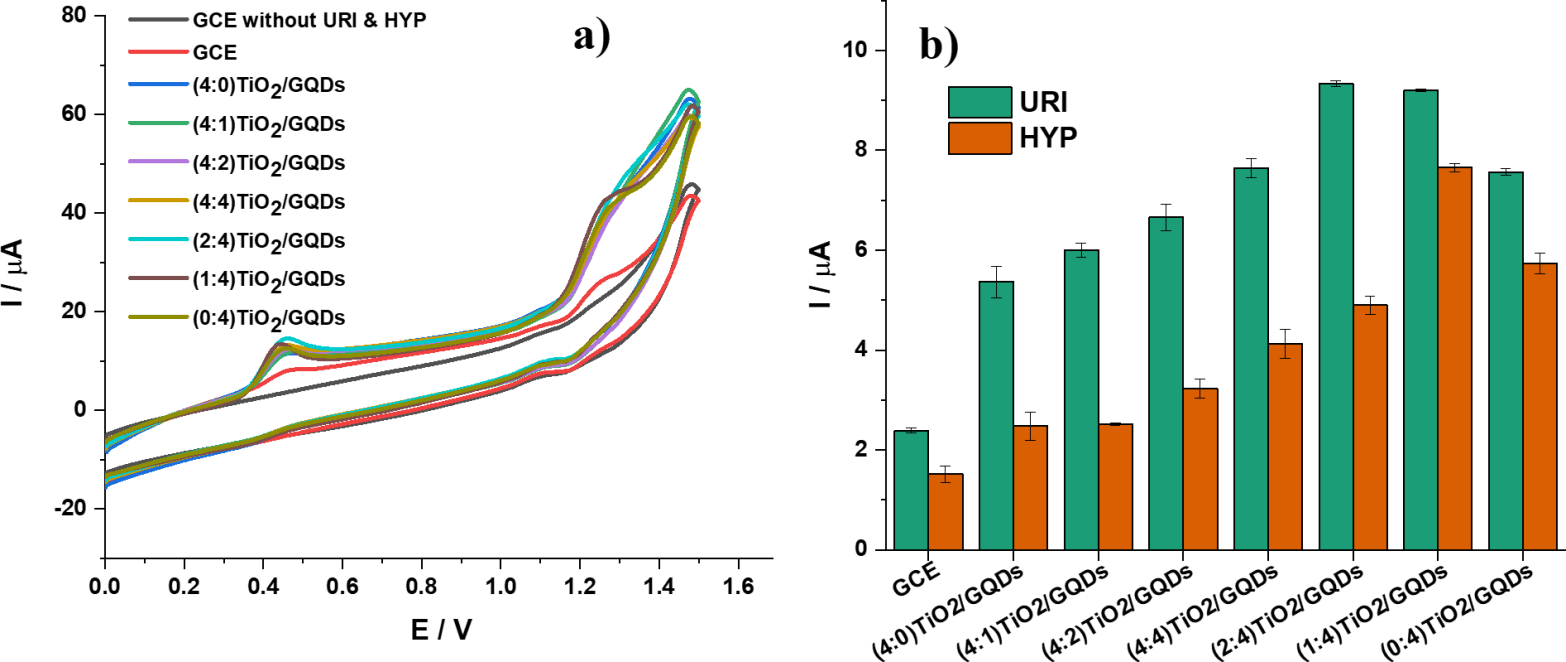
(a) CV curves at different electrodes in a solution (0.05 M BR buffer, pH 4) containing *C*_URI_ = *C*_HYP_ = 25 µM at a potential scan rate *v* = 0.1 V·s^−1^. (b) Oxidation peak current of URI and HYP at different electrodes (the error bar represents the standard deviation of three measurements).

The cyclic voltammetric (CV) behaviors of different electrodes are presented in Figure 5a. The CV curve of the bare GCE shows broad and weak anodic peaks of URI and HYP oxidation. The electrochemical signals are significantly enhanced when the electrode is modified with TiO_2_/GQDs. A well-defined oxidation peak appears at 0.45 V for URI and at 1.26 V for HYP at the TiO_2_/GQDs-GCE. It is notable that the electrochemical response is a function of the TiO_2_/GQDs composition (Figure 5b). The oxidation peak currents of URI and HYP increase with increasing amount of GQDs in TiO_2_/GQDs. However, the peak current of URI reaches its maximum when the GQDs reach 67% in mass (2:4)TiO_2_/GQDs and decreases at higher amounts of GQDs. Meanwhile, the HYP oxidation peak current is highest when the amount of GQDs reaches 80% in mass in (1:4)TiO_2_/GQDs. To balance between the peak currents of URI and HYP, the electrode modified by (1:4)TiO_2_/GQDs was chosen for further investigation. At the (1:4)TiO_2_/GQDs-GCE, the oxidation peak currents for URI and HYP are, respectively, 3.8 times and 5.1 times higher than those obtained at the bare GCE. URI is a weak diprotic acid with p*K*_a1_ = 5.4 and p*K*_a2_ = 9.8 [30]. HYP contains an ionizable group with p*K*_a_ = 8.7 [31]. The improved reversibility and sensitivity of the modified electrode may be due to the electrostatic interaction between positively charged URI and HYP and negatively charged GQD at remaining COO^−^ groups, the complexation of titanium to nitrogen or oxygen, or π–π interactions between the phenyl structure of purine and the planar hexagonal carbon structure of graphene.

#### Effect of pH

**Figure 6:**
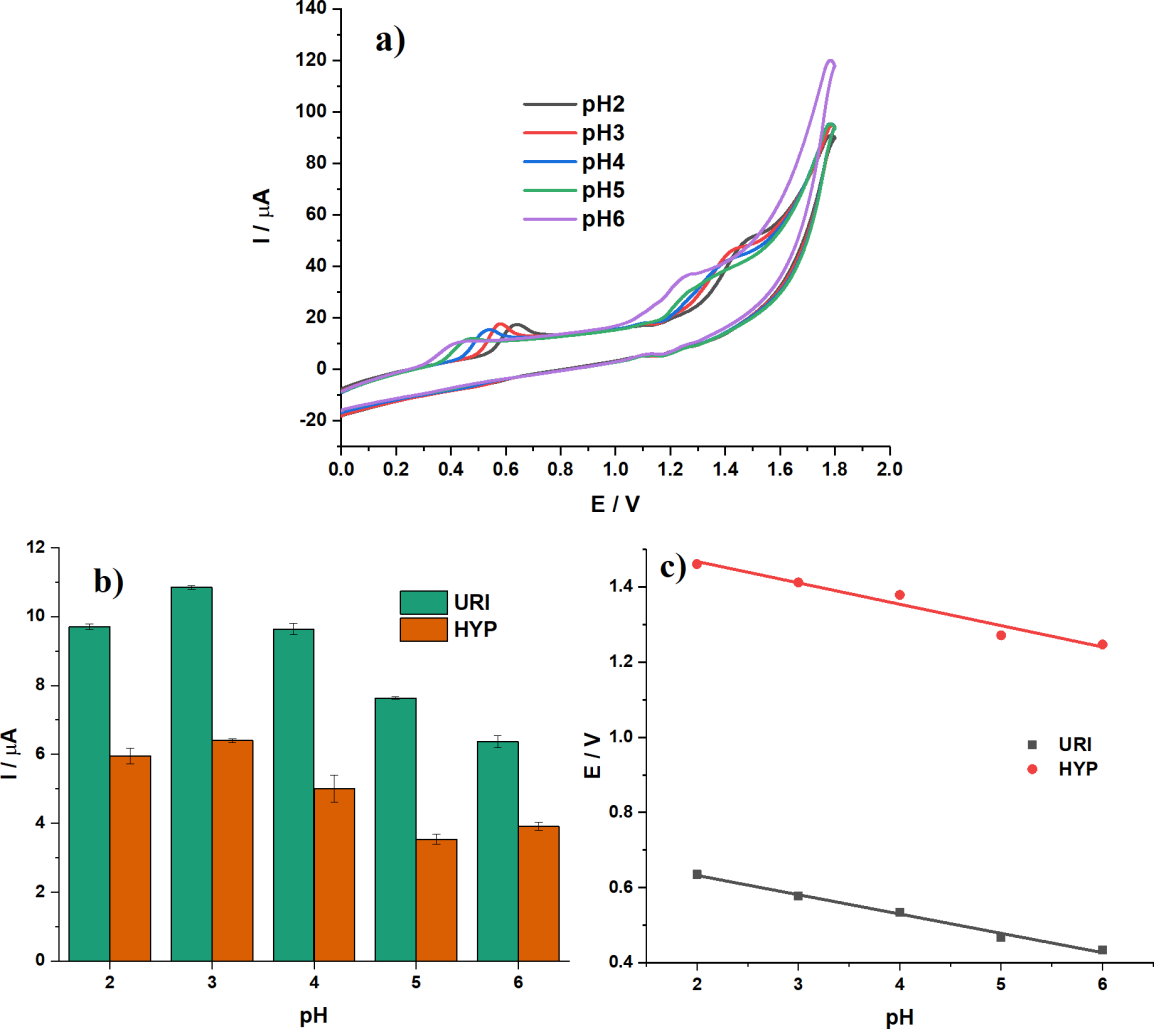
(a) CV curves of URI and HYP at equal concentrations of 25 µM in 0.05 M BR pH 2–6 at the (1:4)TiO_2_/GQDs-GCE; potential scan rate *v* = 0.1 V·s^−1^. (b) Oxidation peak current of URI and HYP at different pH values. (c) Linear plots of peak potential vs pH.

Figure 6a presents the pH dependence of the electrochemical response. The values of *E*_p_ and *I*_p_ vary as a function of pH, indicating that the oxidation process involves the transfer of protons. The oxidation peak current of both analytes peaks at pH 3 then decreases with further increase of pH. Therefore, pH 3 was chosen for further experiments (Figure 6b). At the same time, the peak potential of both analytes shifts towards positive values with a linear relationship described by the following equations (Figure 6c):


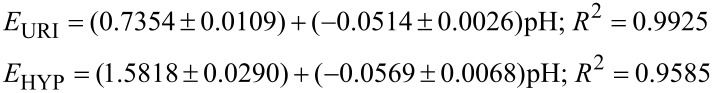


The slopes of the *E*_URI_ vs pH and *E*_HYP_ vs pH plots are close to the theoretical value of the Nernst equation (0.0599), indicating that the oxidation process involves the transfer of an equal number of protons and electrons.

#### Effect of scan rate

**Figure 7:**
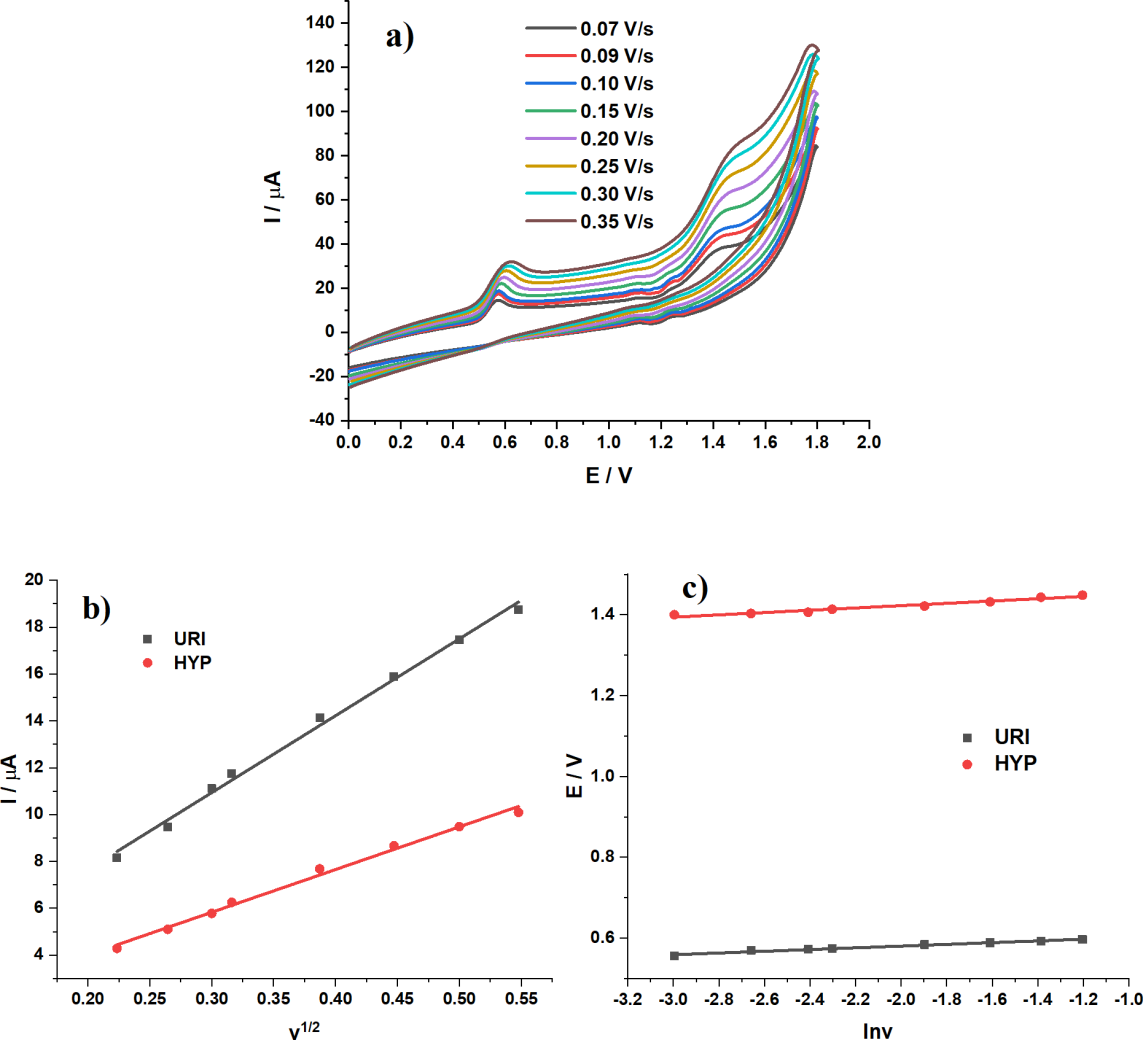
(a) CV curves of (1:4)TiO_2_/GQDs-GCE in 0.05 M BR buffer (pH 3) with *C*_URI_ = *C*_HYP_ = 25 µM; scanning rate *v* = 0.07–0.35 V·s^−1^. (b) Linear curves of peak intensity as a function of *v*^1/2^. (c) Linear curves of peak potential of URI or HYP as a function of ln *v.*

The effect of the scan rate on the electrochemical signals is illustrated in Figure 7a. The linear relationship between peak current and square root of scan rate is presented in Figure 7b with the following linear equations:


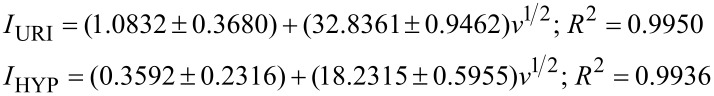


The lines do not pass the origin, indicating that the oxidation process of both URI and HYP are controlled by adsorption processes [32].

The relationship between peak potential and natural logarithm of scan rate is described by the Laviron equation [33] (Figure 7c).


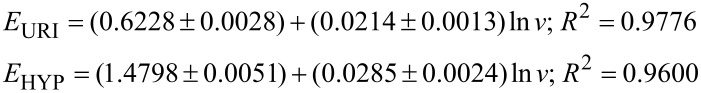


**Scheme 1:**
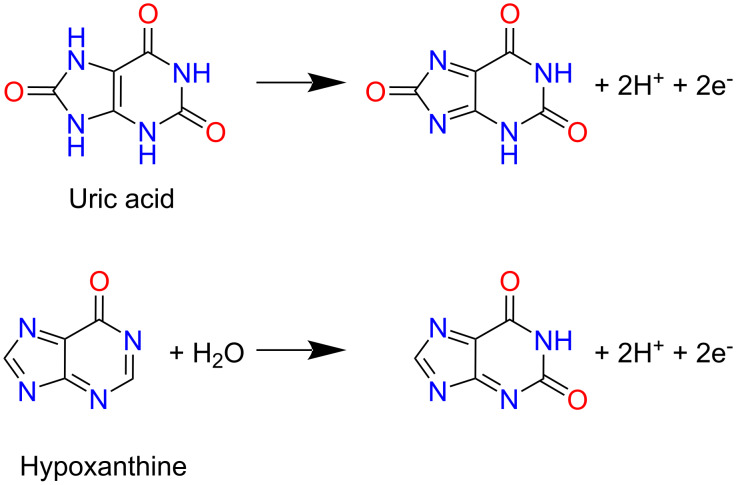
Proposed oxidation mechanisms of URI and HYP at the (1:4)TiO_2_/GQDs-GCE.

The product *n*α was found as 1.2 for URI and 0.9 for HYP. For an irreversible system, α is assumed to be 0.5 [34]; therefore, *n* = 2.4 or 2 for URI and *n* = 1.8 or 2 for HYP, which indicates that the oxidation of URI and HYP involves the transfer of two electrons and two protons. Based on the above observations, plausible mechanisms for the electrooxidation of URI and HYP are proposed (Scheme 1), which are in good agreement with previous reports, where the oxidation of URI generates a bis-imine [35], and the oxidation of HYP forms 6,8-dioxypurine at the N7=C8 double bond [36].

#### Relevant operational parameters

In the present work, the operational parameters accumulation potential (*E*_acc_), accumulation time (*t*_acc_), pulse amplitude (Δ*E*), and potential step (*U*_step_) were optimized. *E*_acc_ does not significantly affect the oxidation signals (*E*_P_ and *I*_P_) of URI and HYP (Supporting Information File 1, Figure S1a,b) when it is varied from −0.2 V to +0.4 V (vs Ag/AgCl|KCl 3 M). Specifically, *E*_P_ and *I*_P_ for the oxidation of URI remain constant at 0.510 ± 0.000 V and 13.43 ± 0.25 μA (*n* = 7), respectively; while the values for HYP are 1.341 ± 0.004 V and 7.204 ± 0.055 μA (*n* = 7), respectively. This shows that the TiO_2_/GQDs material interacts well with URI and HYP during the accumulation step. An *E*_acc_ value of +0.1 V with low relative standard deviation (RSD) values for *I*_P_ of URI and HYP of 0.81% and 2.92%, respectively, was chosen to avoid any potential interference effect in real samples. The variation of the potential scan rate during DPV measurement reveals that the electrochemical reactions of URI and HYP are determined by adsorption. Therefore, even at *t*_acc_ = 0, the *I*_P_ values for URI and HYP are 3.787 ± 0.535 μA (*n* = 3) and 4.193 ± 0.318 μA (*n* = 3), respectively (Supporting Information File 1, Figure S1c,d). This phenomenon shows that URI and HYP quickly adsorb on the surface of the modified electrode at the beginning of the measurement. Therefore, when the accumulation time increases from 5 to 20 s, the *I*_P_ values of URI and HYP are not statistically different. However, it is possible that the high concentration of URI and HYP in the solution might saturate on the modified electrode surface. Therefore, an accumulation time of 15 s was chosen for further studies.

The effect of pulse amplitude (Δ*E*) was also studied (Supporting Information File 1, Figure S2a,b). When Δ*E* gradually increases from 0.04 to 0.08 V, the *I*_P_ of URI gradually increases, reaches the highest value at 0.10 V, and then remains constant up to Δ*E* of 0.12V. In contrast, the *I*_p_ of HYP rises with the increase of Δ*E,* peaks at 0.08 V, and then slightly decreases at higher pulse amplitudes. Furthermore, the HYP oxidation peak broadens with an increase of the peak half-width from 0.167 to 0.180 V as the pulse amplitude increases (Supporting Information File 1, Figure S2a). The oxidation peak of URI shifts positively as the pulse amplitude increases. This behavior might reduce the selectivity of the analytical method. Therefore, a pulse amplitude of 0.08 V was chosen for further studies. *U*_step_ in differential pulse anodic stripping voltammetry affects the potential scan rate and the stripping signal. The influence of *U*_step_ was studied in the range from 0.004 to 0.010 V corresponding to a potential scan rate from 13.3 mV·s^−1^ to 33.3 mV·s^−1^ with *t*_step_ = 0.3 s. It is clear that *U*_step_ slightly changes the oxidation potential of both HYP and URI. Theoretically, increasing the potential step might lead to an increase of *I*_p_. However, a high *U*_step_ might also trigger the oxidation of other substances with similar oxidation potential, which greatly affects the simultaneous quantification of substances with stripping peaks close to each other. Therefore, a *U*_step_ value of 0.008 V was chosen for further studies.

#### Linear range and limit of detection

**Figure 8:**
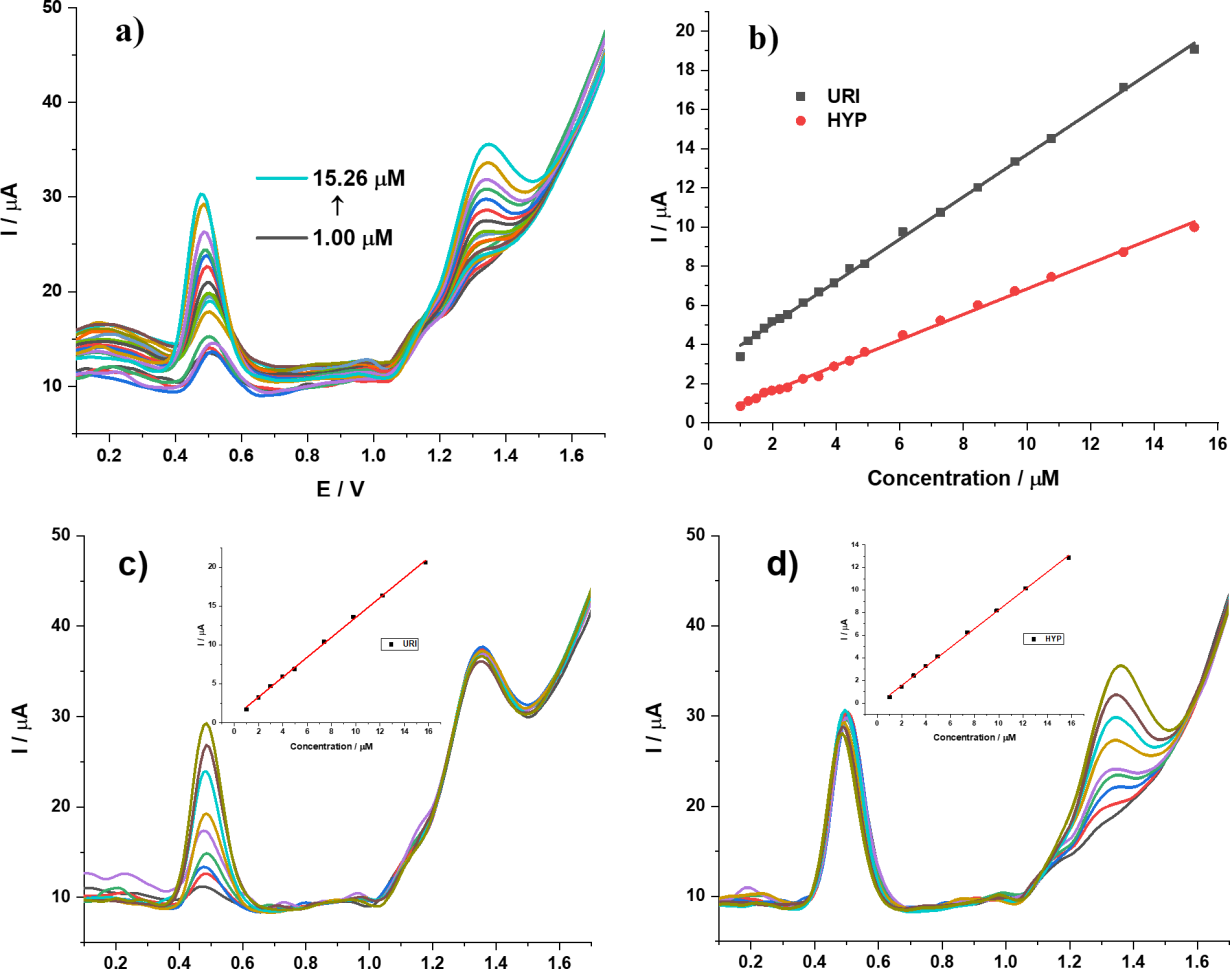
DPV curves at the (1:4)TiO_2_/GQDs-GCE. (a) 0.05 M BR pH 3 with *C*_URI_ = *C*_HYP_ = 1.00–15.26 µM. (b) Linear plots of peak current vs URI and HYP concentration. (c) 0.05 M BR buffer (pH 3) with *C*_URI_ = 1.00–15.74 µM, *C*_HYP_ = 15.74 µM, the inset presents a linear plot of peak current vs URI concentration. (d) 0.05 M BR buffer (pH 3) with *C*_HYP_ = 1.00–15.74 µM, *C*_HYP_ = 15.74 µM, the inset presents a linear plot of peak current vs HYP concentration.

DPV curves for mixtures containing equal concentrations of URI and HYP are shown in Figure 8a. The peak current increases linearly when the concentration of both analytes increases from 1.00 to 15.26 µM (Figure 8b). The regression equations are as follows:


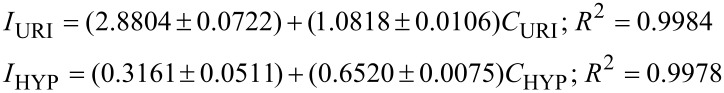


The limit of detection (LOD) is described as 3*S*/*M*, where *S* is the standard deviation and *M* is the slope obtained from the calibration plot. The LOD values are 0.58 µM for URI and 0.68 µM for HYP. The cross-effect is a key factor for the simultaneous determination of analytes in a mixture. Figure 8c presents the effect of HYP concentration, which was kept constant at 15.74 μM, on the peak current intensity of different concentrations of URI. A linear response (the inset) is still found in the range of 1.00–15.74 μM with the regression equation of *I*_URI_ = (0.7538 ± 0.1356) + (1.2783 ± 0.0166)*C*_URI_ (*R*^2^ = 0.9988) (LOD_URI_ = 0.61 µM). A similar behavior can be observed for the oxidation of an increasing amount of HYP (1.00–15.74 μM) (inset) in the presence of 15.74 μM URI. A linear calibration curve with a regression equation of *I*_HYP_ = (−0.1160 ± 0.0830) + (0.8376 ± 0.0102)*C*_HYP_ (*R*^2^ = 0.9990) with LOD_HYP_ = 0.57 μM (Figure 8d) was found. These results indicate that the determination of URI in the presence of HYP and vice versa at the TiO_2_/GQDs-GCE can be achieved with acceptable selectivity and sensitivity. A comparison with previously reported data is shown in Table 1. It is noticed that the performance of TiO_2_/GQDs-GCE is better than or comparable with other electrodes with low detection limit.

**Table 1:** Comparison of URI and HYP determination at the TiO_2_/GQDs-GCE with the literature.

Electrode	Method	Linear range (µM)		LOD (µM)		Applicability	Ref
		URI	HYP	URI	HYP		

Nf–{RuDMSO–Cl–H_2_O}–MME/GCE	SWV	100–700	50–300	0.372	2.37	human urine samples	[37]
Co_2_Fe_2_O_4_/rGO/GCE	DPV	2–10	2–10	0.767	0.506	urine samples	[38]
GCE/rGO/CS/Cr_2_O_3_/GCE	DPV	10–500	2–300	0.8	0.85	fish meat	[39]
PDAox–PTCA/GCE	DPV	1.8–238	3.8–293	1.50	1.25	biologically species	[40]
β-cyclodextrin/GCE	DPV	10–225	10–270	5	5	human urine samples	[41]
TiO_2_/GQDs	DPV	1.0–15.3	1.0–15.3	0.58	0.68	urine samples	this work

#### Repeatability, stability and reproducibility

**Figure 9:**
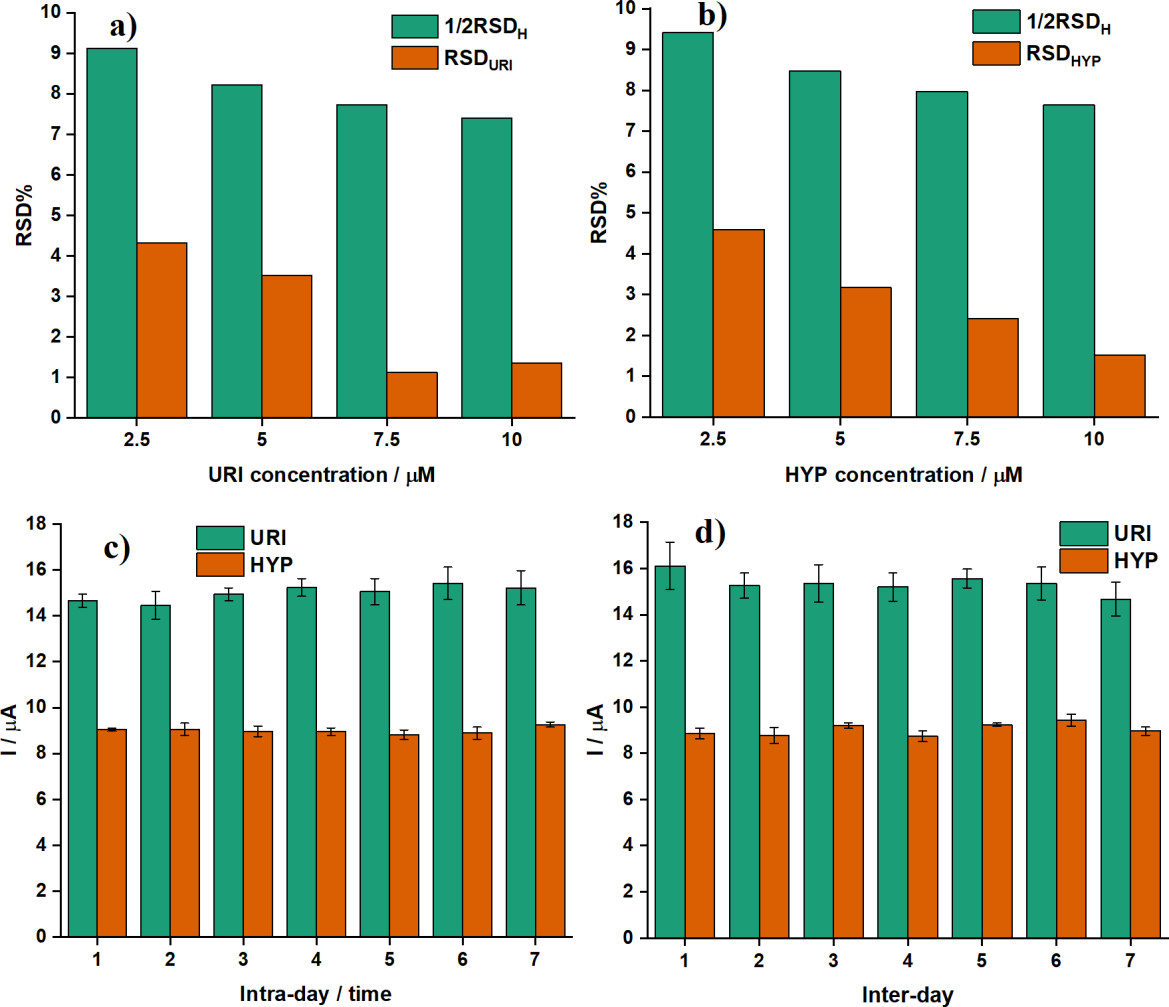
The RSD of ten replicate measurements of (a) URI and (b) HYP at the (1:4)TiO_2_/GQDs-GCE in 0.05 M BR buffer pH 3 with *C*_URI_ = *C*_HYP_ = 2.5, 5.0, 7.5, and 10 µM. (c) *I*_p_ of URI and HYP (*C*_URI_ = *C*_HYP_ = 12 µM) in 0.05 M BR buffer pH 3 at seven different (1:4)TiO_2_/GQDs-GCEs fabricated using the same procedure. (d) *I*_p_ of URI and HYP (*C*_URI_ = *C*_HYP_ = 12 µM) in 0.05 M BR buffer pH 3 at the (1:4)TiO_2_/GQDs-GCE measured once a week.

The parameters for validity of the proposed approaches are the within-run precision (repeatability), reproducibility, and the within-laboratory precision (long term-stability). The repeatability of the method was evaluated by comparing the RSD of the measurement to the RSD of the Horwitz function (RSD_H_). If RSD is less than half of *R*SD_H,_ the repeatability is acceptable. The repeatability of this method was determined by analyzing ten replicate measurements on four different mixtures containing equal concentrations of URI and HYP (2.5, 5.0, 7.5, and 10 μM). The results show that all RSDs are smaller than 5% and 0.5·RSD_H_ (Figure 9a,b) indicating an acceptable repeatability. The reproducibility was estimated by measuring electrochemical signals of URI and HYP with seven modified electrodes fabricated using the same procedure. The peak currents (three consecutive measurements) of equal concentrations of URI and HYP (*C*_URI_ = *C*_HYP_ = 12 µM) at each modified electrode are given in Figure 9c. The peak currents were found as *I*_URI_ = 14.99 ± 0.34 (µA) with RSD = 2.26% and *I*_HYP_ = 9.00 ± 0.14 (μA) with RSD = 1.59%. An RSD smaller than 5% indicates that the proposed technique is reproducible. Stability is a critical characteristic of electrochemical sensors; it can be determined by comparing electrochemical signals from a single electrode, which is kept at 4 °C in the BR buffer pH 3 between the measurements, for a definite period. DPV measurements were periodically performed once a week. Peak currents with RSD smaller than 5% were found as *I*_URI_ = 15.36 ± 0.43 (µA) (RSD = 2.82%) and *I*_HYP_ = 9.03 ± 0.26 (µA) (RSD = 2.93%) indicating good long-term stability of the (1:4)TiO_2_/QGDs-GCE (Figure 9d).

**Figure 10:**
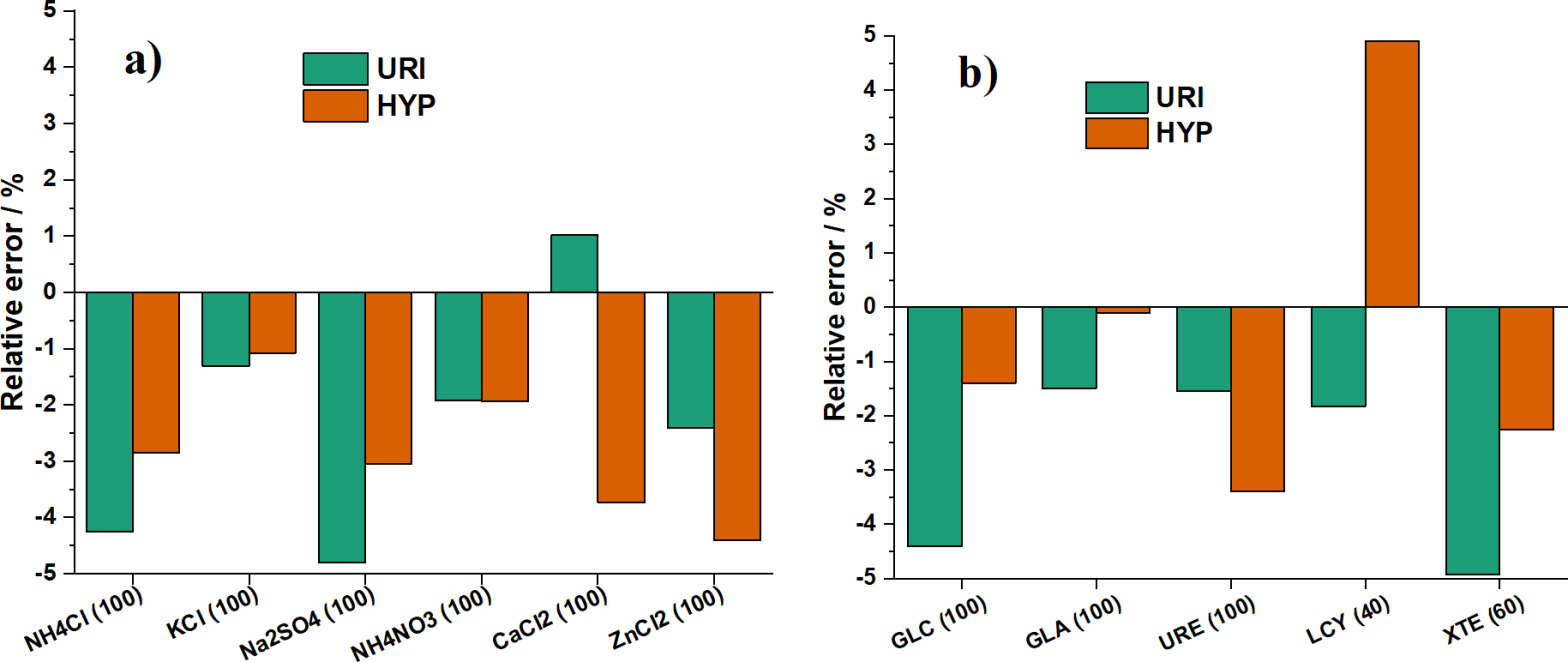
Effect of possible interferents. (a) Inorganic compounds and (b) organic compounds.

In order to study the selectivity of the (1:4)TiO_2_/GQDs-modified electrode, common co-existing substances, namely, NH_4_Cl, KCl, Na_2_SO_4_, NH_4_NO_3_, CaCl_2_, ZnCl_2_, glucose (GLC), glutamic acid (GLA), urea (URE), ʟ-cysteine (LCY) and xanthine (XTE), have been added to the analysis mixture containing URI (10 μM) and HYP (10 μM) to investigate the interference effect on the electrochemical responses (Figure 10 and Supporting Information File 1, Table S1). The results show that, except LCY and XTE affecting the measurement of URI and HYP at 40 and 60-fold excess, the other possible interferents exhibit no interference effect (with less than ±5% relative error) at 100-fold excess. This indicates that the designed modified electrode is a highly selective sensor for URI and HYP.

A human urine sample was used as real sample to investigate the reliability of the proposed method. The urine sample was diluted 130 times with 0.05 M BR buffer pH 3 before measurement. No other pretreatment process was required. The standard addition method was employed to test the recovery. The results show an exceptional recovery range from 96.57% to 103.58%, which clearly indicates the applicability and reliability of the proposed method (Table 2).

**Table 2:** Determination of URI and HYP in real samples (*n* = 5) with the (1:4)TiO_2_/GQDs-GCE.

Sample	Analyte	Content ± SD (µM/)	Spiked (µg)	Found* ± SD (µg)	Recovery (%)

human urine 1	URI	2.633 ± 0.072	0.841	0.814 ± 0.029	96.84
	HYP	—	1.361	1.332 ± 0.030	97.88
human urine 2	URI	2.793 ± 0.066	0.841	0.860 ± 0.027	102.30
	HYP	—	1.361	1.341 ± 0.018	98.53
human urine 3	URI	4.493 ± 0.117	0.841	0.812 ± 0.023	96.57
	HYP	—	1.361	1.410 ± 0.030	103.58

## Conclusion

TiO_2_/GQDs were prepared from peroxo titanium complexes, and GQDs were derived from ground coffee as precursor, which offers several advantages such as easy dispersion and functionalization during the synthesis. A simple strategy employing TiO_2_/GQDs was used to fabricate a sensitive voltammetric sensor for the determination of URI and HYP in real samples. The decoration of nanoscale TiO_2_ with GQDs can provide excellent electrode modification because of the combination of enlarged active surface area and strong adsorptive capability of the nanomaterials. Low LODs, high reproducibility, and simple procedures for surface modification and analysis can be considered as the advantages of the proposed DPV method.

## Supporting Information

File 1 Additional figures and tables.

## Data Availability

All data that supports the findings of this study is available in the published article and/or the supporting information to this article.
