# Supplementary material for: Simultaneous electrochemical determination of uric acid and hypoxanthine at a TiO2/graphene quantum dot-modified electrode
Source: Beilstein J Nanotechnol. 2024 Jun 20;15:719–32. doi: 10.3762/bjnano.15.60 (PMC11196949; doi:10.3762/bjnano.15.60)
Supplement: File 1 — Additional figures and tables. [file Beilstein_J_Nanotechnol-15-719-s001.pdf]

BEILSTEIN JOURNAL OF NANOTECHNOLOGY

Supporting Information

for

Simultaneous electrochemical determination of uric acid and hypoxanthine at a TiO_2/graphene quantum dot-modified electrode

Vu Ngoc Hoang, Dang Thi Ngoc Hoa, Nguyen Quang Man, Le Vu Truong Son, Le Van Thanh Son, Vo Thang Nguyen, Le Thi Hong Phong, Ly Hoang Diem, Kieu Chan Ly, Ho Sy Thang and Dinh Quang Khieu

Beilstein J. Nanotechnol. **2024,** *15,* 719–732. doi:10.3762/bjnano.15.60

Additional figures and tables

License and Terms: This is a supporting information file under the terms of the Creative Commons Attribution License (https://creativecommons.org/licenses/by/4.0). Please note that the reuse, redistribution and reproduction in particular requires that the author(s) and source are credited and that individual graphics may be subject to special legal provisions.

The license is subject to the *Beilstein Journal of Nanotechnology* terms and conditions: (https://www.beilstein-journals.org/bjnano/terms)

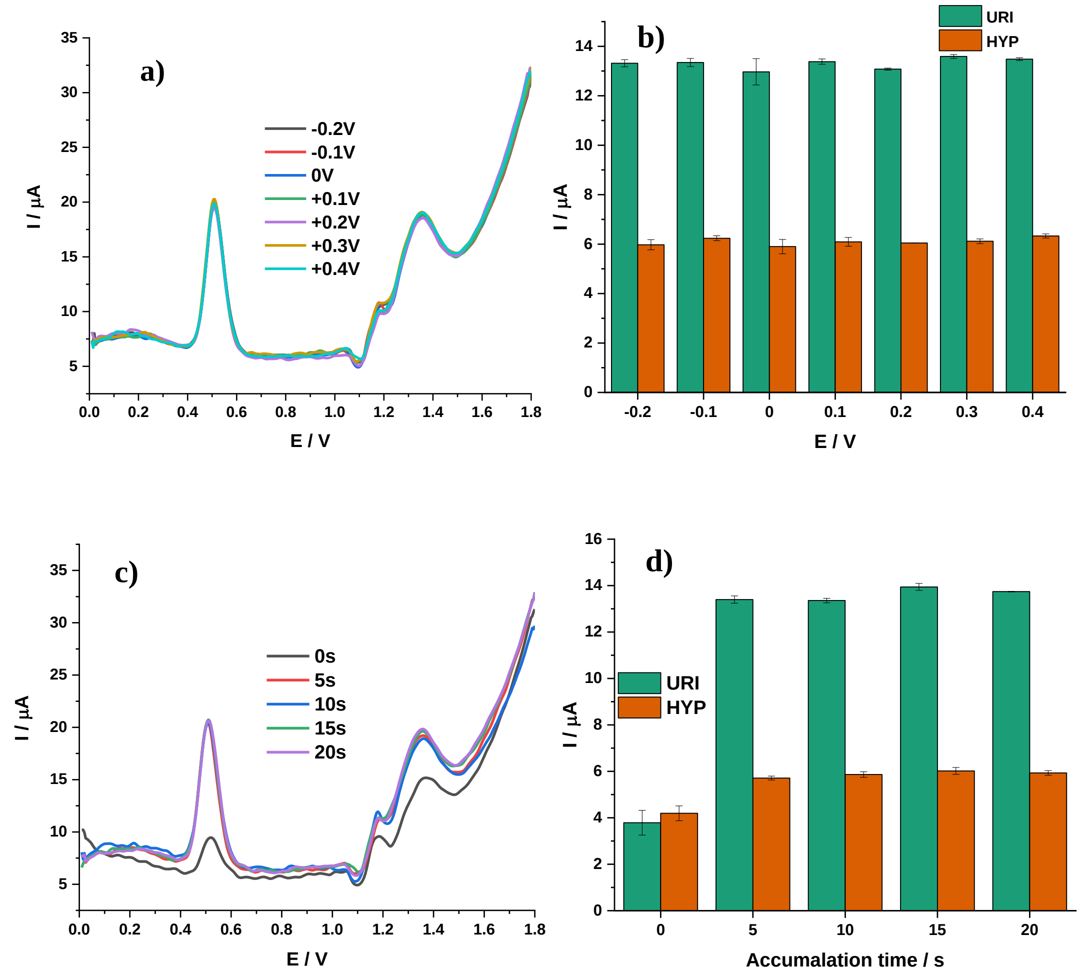

Figure S1: a) DPV curves of URI and HYP ($C_{URI} = C_{HYP}$ = 25 µM) in 0.05 M BRS (pH 3) at (1:4)TiO_2/GQD/GCE at various accumulation potentials (in the range from −0.2 V to +0.4 V) and b) the dependence of peak current, I_p on accumulation potential; c) DPV curves of URI and HYP ($C_{URI} = C_{HYP}$ = 25 µM) in 0.05 M BRS (pH 3) at (1:4)TiO_2/GQD/GCE with various accumulation time (in the range from 0 to 20 s) at accumulation potential of −0.1V and d) the dependence of I_p on accumulation time.

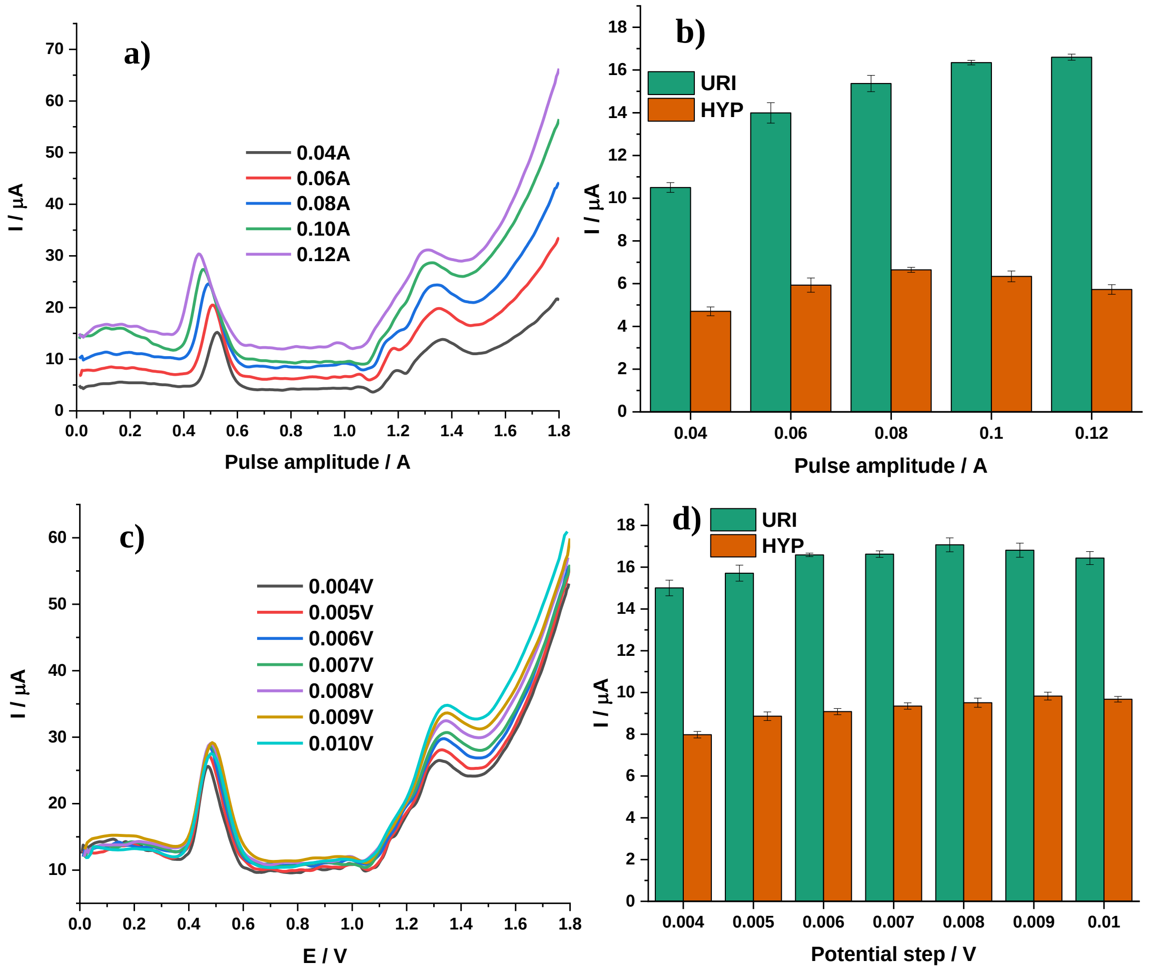

Figure S2: a) DPV curves of URI and HYP (C_{URI} = C_{HYP} = 25 µM) at (1:4)TiO_2/GQD/GCE in BRS 0.05 M (pH 3) with various pulse amplitudes (0.04 to 0.12A) at the −0.1 V accumulation potential and 15 s accumulation time and b) the dependence of peak currents on pulse amplitude; c) DPV curves of URI and HYP (C_{URI} = C_{HYP} = 25 µM) at (1:4)TiO_2/GQD/GCE in BRS 0.05 M (pH 3) with various potential from 0.004 to 0.010 V at −0.1 V accumulation potential, 15 s accumulation time and 0.10 A pulse amplitude and d) the dependence of peak currents on potential step.

Table S1: Effect of some interferents on peak current of URI and HYP.

**1. NH_4Cl**							
**C_{NH4Cl}/C_{URI}**	**Ip (µA)**	**RSD (%)**	**Re (%)**	**C_{NH4Cl}/C_{HYP}**	**Ip (µA)**	**RSD (%)**	**Re (%)**
0	11.66	2.53	0.00	0	8.11	2.41	0.00
10	11.91	2.34	2.17	10	8.34	2.01	2.88
40	11.75	1.19	0.77	40	8.24	2.14	1.58
60	11.52	1.82	-1.18	60	8.12	2.48	0.20
80	11.53	1.85	-1.16	80	8.11	1.02	0.04
100	11.17	1.29	-4.25	100	7.88	1.25	-2.86
**2. KCl**							
**C_{KCl}/C_{URI}**	**Ip (µA)**	**RSD (%)**	**Re (%)**	**C_{KCl}/C_{HYP}**	**Ip (µA)**	**RSD (%)**	**Re (%)**
0	11.10	1.82	0.00	0	7.88	1.25	0.00
10	11.32	4.62	1.98	10	7.86	0.81	-0.25
40	11.40	3.35	2.70	40	7.81	1.00	-0.89
60	11.35	1.68	2.21	60	7.81	2.90	-0.83
80	11.02	1.89	-0.72	80	7.99	0.29	1.50
100	10.96	4.32	-1.31	100	7.79	1.82	-1.08
**3. Na_2SO_4**							
**C_{Na2SO4}/C_{URI}**	**Ip (µA)**	**RSD (%)**	**Re (%)**	**C_{Na2SO4}/C_{HYP}**	**Ip (µA)**	**RSD (%)**	**Re (%)**
0	11.19	3.95	0.00	0	7.79	2.94	0.00
10	11.02	1.66	-1.52	10	7.89	1.10	1.28
40	10.80	2.16	-3.49	40	7.68	2.09	-1.45
60	11.07	1.61	-1.04	60	7.89	1.59	1.24
80	10.90	1.20	-2.64	80	7.67	1.97	-1.54
100	10.65	3.62	-4.80	100	7.55	1.03	-3.05
**4. NH_4NO_3**							
**C_{NH4NO3}/C_{URI}**	**Ip (µA)**	**RSD (%)**	**Re (%)**	**C_{NH4NO3}/C_{HYP}**	**Ip (µA)**	**RSD (%)**	**Re (%)**
0	10.58	4.18	0.00	0	7.59	1.19	0.00
10	10.54	2.12	-0.38	10	7.61	2.17	0.30
40	10.49	2.68	-0.85	40	7.45	1.12	-1.78

60	10.45	3.86	-1.16	60	7.51	0.67	-1.09
80	10.37	4.68	-1.94	80	7.41	2.29	-2.30
100	10.37	4.57	-1.91	100	7.44	2.11	-1.94
**5. $CaCl_2$**							
**C_{CaCl2}/C_{URI}**	**Ip (μA)**	**RSD (%)**	**Re (%)**	**C_{CaCl2}/C_{HYP}**	**Ip (μA)**	**RSD (%)**	**Re (%)**
0	10.15	2.63	0.00	0	7.43	1.93	0.00
10	10.34	2.54	1.82	10	7.50	1.47	0.94
40	10.40	1.71	2.46	40	7.40	0.72	-0.40
60	10.12	2.88	-0.32	60	7.14	1.90	-3.84
80	9.99	0.26	-1.58	80	7.32	0.76	-1.45
100	10.25	0.95	1.02	100	7.15	1.19	-3.74
**6. $ZnCl_2$**							
**C_{ZnCl2}/C_{URI}**	**Ip (μA)**	**RSD (%)**	**Re (%)**	**C_{ZnCl2}/C_{HYP}**	**Ip (μA)**	**RSD (%)**	**Re (%)**
0	10.25	1.02	0.00	0	7.25	1.08	0.00
10	9.96	3.20	-2.84	10	7.21	1.40	-0.63
40	9.86	2.98	-3.81	40	7.06	1.91	-2.70
60	10.14	1.12	-1.07	60	7.12	0.86	-1.87
80	9.91	2.80	-3.29	80	7.22	1.53	-0.49
100	10.00	7.35	-2.42	100	6.93	3.56	-4.40
**7. Glucose**							
**C_{GLC}/C_{URI}**	**Ip (μA)**	**RSD (%)**	**Re (%)**	**C_{GLC}/C_{HYP}**	**Ip (μA)**	**RSD (%)**	**Re (%)**
0	10.00	7.37	0.00	0	6.97	2.91	0.00
10	9.74	2.73	-2.55	10	6.93	2.38	-0.54
40	9.72	3.45	-2.78	40	7.07	1.10	1.48
60	9.78	1.90	-2.18	60	6.85	1.55	-1.69
80	9.53	1.86	-4.63	80	6.89	2.12	-1.18
100	9.56	4.38	-4.40	100	6.87	1.42	-1.40
**8. Glutamic acid**							
**C_{GLA}/C_{URI}**	**Ip (μA)**	**RSD (%)**	**Re (%)**	**C_{GLA}/C_{HYP}**	**Ip (μA)**	**RSD (%)**	**Re (%)**
0	9.50	4.06	0.00	0	6.63	3.02	0.00

10	9.53	1.71	0.32	10	6.77	1.56	2.11
40	9.59	1.85	1.02	40	6.84	1.33	3.12
60	9.44	1.47	-0.57	60	6.85	4.09	3.32
80	9.44	3.60	-0.60	80	6.71	1.82	1.21
100	9.36	2.49	-1.49	100	6.62	2.05	-0.11
**9. Urea**							
**C_{URE}/C_{URI}**	**Ip (μA)**	**RSD (%)**	**Re (%)**	**C_{URE}/C_{HYP}**	**Ip (μA)**	**RSD (%)**	**Re (%)**
0	9.38	2.31	0.00	0	6.62	2.05	0.00
10	9.30	2.48	-0.82	10	6.56	1.67	-0.99
40	9.28	2.75	-1.05	40	6.64	0.67	0.19
60	9.12	1.04	-2.75	60	6.47	4.17	-2.30
80	9.36	1.74	-0.21	80	6.52	3.50	-1.59
100	9.23	0.91	-1.55	100	6.40	1.46	-3.40
**10. L-cysteine**							
**C_{LCY}/C_{URI}**	**Ip (μA)**	**RSD (%)**	**Re (%)**	**C_{LCY}/C_{HYP}**	**Ip (μA)**	**RSD (%)**	**Re (%)**
0	9.24	0.85	0.00	0	6.38	0.66	0.00
10	9.49	1.56	2.76	10	6.49	3.83	1.71
40	9.07	1.95	-1.82	40	6.69	2.12	4.90
60	8.95	2.52	-3.14	60	7.15	1.92	**12.15**
80	9.02	4.01	-2.29	80	6.99	1.19	**9.55**
100	8.83	3.74	-4.41	100	7.23	1.99	**13.37**
**11. Xanthine**							
**C_{XTE}/C_{URI}**	**Ip (μA)**	**RSD (%)**	**Re (%)**	**C_{XTE}/C_{HYP}**	**Ip (μA)**	**RSD (%)**	**Re (%)**
0	8.58	3.37	0.00	0	6.32	1.43	0.00
10	8.30	1.35	-3.25	10	6.35	4.98	0.47
40	8.19	2.12	-4.57	40	6.18	4.21	-2.21
60	8.16	2.77	-4.92	60	6.17	2.43	-2.26
80	7.50	5.86	**-12.57**	80	6.01	2.02	-4.82
100	7.26	8.60	**-15.41**	100	6.04	1.73	-4.35
